# How to use a rotating ring-disc electrode (RRDE) subtraction method to investigate the electrocatalytic oxygen reduction reaction?†

**DOI:** 10.1039/d2cy01744j

**Published:** 2022-12-23

**Authors:** Angelina Kerschbaumer, Dominik Wielend, Elisabeth Leeb, Corina Schimanofsky, Nadine Kleinbruckner, Helmut Neugebauer, Mihai Irimia-Vladu, Niyazi Serdar Sariciftci

**Affiliations:** a Linz Institute for Organic Solar Cells (LIOS), Institute of Physical Chemistry, Johannes Kepler University Linz Altenberger Straße 69 4040 Linz Austria elisabeth.leeb@jku.at

## Abstract

When studying electrochemical oxygen reduction reactions in homogeneous media, special attention must be given to the significant background activity present with conventional electrode materials. The intrinsic electrocatalytic activity of different materials can be investigated using complementary methods, such as the rotating ring-disc electrode (RRDE) technique and chronoamperometric electrolysis with product quantification. This report presents a detailed investigation of the electrocatalytic ability of hydroxy anthraquinone derivatives and riboflavin towards hydrogen peroxide (H_2_O_2_) production *via* a novel RRDE subtraction method together with chronoamperometric electrolysis. Qualitative trends linking the two methods were obtained, such as a higher excess current correlating with both higher productivity and selectivity. As such, a valuable tool is provided to increase the understanding of the electrocatalytic ability of homogeneous solutions toward improving the oxygen reduction reaction.

## Abstract 2

The intrinsic electrocatalytic activity of different homogeneous materials can be investigated using complementary methods, such as rotating ring-disc electrode (RRDE) and chronoamperometric electrolysis.

## Graphical abstract

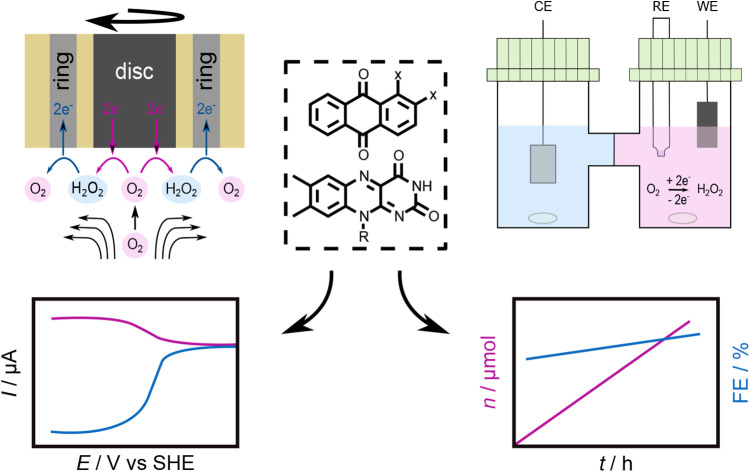

## 1. Introduction

During the last few years, great effort in renewable energy supply (like solar energy or wind energy) has been observed, increasing the demand for energy storage solutions of such renewable electricity.^1–4^ Although nowadays mainly inorganic materials are used in energy storage technologies such as electrolysers or batteries, in the last few years, emerging organic materials for energy storage applications have been steadily growing in interest.^5–8^ Organic compounds like quinones are renowned electrocatalysts already discovered decades ago, especially in the field of electrochemical oxygen (O_2_) reduction to hydrogen peroxide (H_2_O_2_),^9–11^ which is a promising compound to be used as chemical energy storage.^12,13^ However, modifications of pure carbon like nanotubes^14^ or doped carbon structures,^15–18^ molecular systems like quinacridone and epindolidione,^19^ or trioxotriangulene^20^ and conductive polymers^21^ like polyaniline,^22–24^ polypyrrole^22,23^ and poly(3,4-ethylenedioxythiophene)^25,26^ are reported as electrocatalysts for H_2_O_2_ production.

Numerous studies regarding covalently attached^27,28^ or adsorbed quinones^29^ exist. However, these reports mainly report purely kinetic data rather than catalytic parameters like production rate or turnover numbers. It is well-known that the type of immobilisation of the catalyst for heterogeneous catalysis strongly affects the electrochemical performance.^30–32^ Furthermore, homogeneously dissolved quinones have also been investigated for their electrocatalytic properties towards O_2_ reduction^33,34^ and reported as redox mediators for photoelectrochemical H_2_O_2_ production cells.^35,36^

Bearing these issues in mind, this work aims to move one step further in characterising quinones as electrocatalysts for O_2_ towards H_2_O_2_ reduction. Firstly, we used differently substituted quinones to gain insight into structure–property relations. Secondly, in addition to mechanistic-kinetic data, real quantification of H_2_O_2_ produced during electrolysis operations is crucial for future technical applications.

However, the fact that nearly all conventional electrode materials show an oxygen reduction reaction (ORR) behaviour complicates homogeneous electrocatalytic investigations. Many commonly used metal electrodes possess a dominating tendency towards a four-electron reduction of O_2_ to water. However, carbon-based electrodes were identified to favour the two-electron pathway towards H_2_O_2_, and are thus prime candidates for studying the electrocatalytic ability of organic materials towards the ORR.^23,29,37,38^ This tendency of carbon-based electrodes for electrochemical H_2_O_2_ production has already been known since the report of Berl^39^ in 1939 and later identified as surface-carbonyl groups acting as active sites.^40,41^ Specifically, this intrinsic electrocatalytic activity of the electrode material might overlap with the potential range of organic materials like quinones, which requires special consideration of this background activity.^42^

As the development of the rotating ring-disc electrode (RRDE) method has been closely related to ORR studies since the beginning,^43^ currently, the RRDE method is still an important tool in ORR research.^10,18,44,45^ However, overlapping reduction and oxidation potentials of the catalysts, products such as H_2_O_2_, and the properties of the electrode material itself impede such RRDE investigations. A recent report by the group of Marc Koper described an RRDE method for heterogeneous carbon dioxide (CO_2_) reduction, how to distinguish contributions of hydrogen formation and carbon monoxide formation by electrode and potential modifications.^46^ Recently, we have followed this subtraction approach, modified it for homogeneous electrocatalytic studies for H_2_O_2_ production and demonstrated its feasibility for a known material, anthraquinone sulfonate (AQS).^42^ Following this strategy, the present work aims at a comparative investigation of six differently substituted anthraquinones with this RRDE subtraction method. To better understand the subtraction results, a direct comparison of this RRDE method with the quantified catalytic electrolysis towards H_2_O_2_ is performed.

In addition to quinones, flavines are also reported as promising electrocatalysts for O_2_ to H_2_O_2_ reduction both in homogeneous solution^47,48^ and in heterogeneous catalysis.^49–51^ Recently, we have investigated the homogeneous ORR of the naturally-occurring vitamin B_2_, riboflavin (RF), on various carbon electrode materials and now included RF as a catalyst molecule in this comparative study.^52^

This work aims to demonstrate the applicability of the novel RRDE subtraction method for homogeneously dissolved electrocatalysts for the ORR as a fast yet powerful tool for electrocatalytic studies.

## 2. Experimental section

### 2.1 RRDE and RRDE-subtraction method

RRDE experiments were performed using an IPS Jaissle Bipotentiostat-Galvanostat PGU BI-1000 equipped with an IPS Rotator 2016 and an IPS PI-ControllerTouch. An RRDE consisting of a GC disc (*∅* = 5 mm) and a platinum (Pt) ring (*∅* = 7 mm) in polyether ether ketone was used as the working electrode (WE). The GC/Pt-RRDE was polished using deagglomerated alumina (Al_2_O_3_) pastes (Buehler Micropolish II) with particle sizes of 1.0, 0.3 and 0.05 μm in decreasing order. The electrode was sonicated in 18 MΩ water as well as in isopropanol (VWR chemicals) between the polishing steps. Furthermore, a platinised counter electrode (CE) and a commercial Ag/AgCl (3 M KCl) (Messtechnik Meinsberg) reference electrode (RE) inside a Luggin capillary were used. Before every RRDE measurement, the cell was purged with N_2_ and O_2_ for 1 h each.

For linear sweep voltammetry (LSV), a scan rate of 10 mV s^−1^ was used. The required ring potential was determined to enable the detection of H_2_O_2_ through re-oxidation while avoiding the oxidation of the electrocatalytic compound, as can be seen in Fig. S1.† Unless stated otherwise, a constant ring potential of 0.26 V *vs.* standard hydrogen electrode (SHE) was applied.

As already reported,^10,18^ the difference in the concentration of the electroactive species between the disc and the ring electrode can be assessed using the collection efficiency (*N*_max_). *N*_max_ was determined through calibration with K_3_[Fe(CN)_6_] (Merck) and calculated according to eqn (1) using the limiting ring current (*I*_R,lim_) as well as the limiting disc current (*I*_D,lim_). These currents are obtained once the system is under steady-state conditions at potentials more negative than −0.3 V *vs.* SHE.1
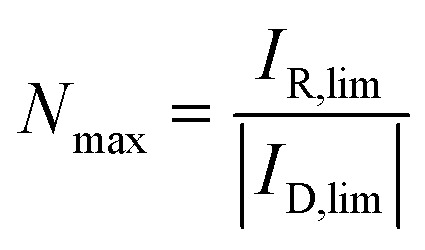
The resulting calibration graph at various rotation speeds can be found in Fig. S2.† Using *N*_max_, the faradaic efficiency (FE) of RRDE experiments can be calculated from the ring current (*I*_R_) and the disc current (*I*_D_) according to eqn (2).2
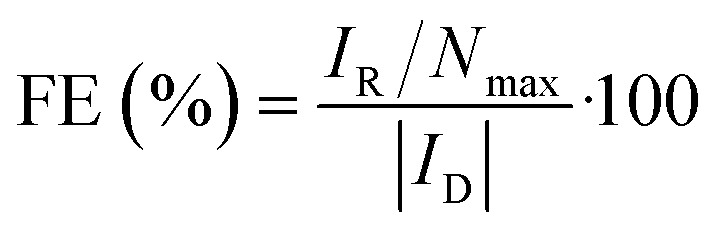
The RRDE subtraction method used in this work has been established by Wielend *et al.*^42^ and similarly reported by Goyal *et al.*^46^ As the total current of the system (*I*_cat/O_2__) consists of the reductive current of O_2_ (*I*_GC/O_2__) as well as the reductive current of the catalyst (*I*_cat/N_2__), an excess current defining the electrocatalytic capability of the catalyst towards the ORR can be calculated according to eqn (3).

3 *I*_excess_ = *I*_cat/O_2__ − (*I*_GC/O_2__ + *I*_cat/N_2__)

Using eqn (2), an excess efficiency can be calculated.

### 2.2 Electrochemical characterisation

1 × 4 cm glassy carbon (GC) plates (Alfa Aesar) with a thickness of 2 mm were polished using the same Al_2_O_3_ pastes as before, with decreasing particle sizes. Prior to the change of polishing paste, the plates were rinsed and sonicated in 18 MΩ water and isopropanol for 10 minutes each. The electrodes were electrochemically activated in 0.5 M sulfuric acid (J. T. Baker) before being used in electrocatalytic experiments. Thus, the potential was swept between 1.5 V and −1.0 V *vs.* Ag/AgCl (3 M KCl) for 30 cycles at a scan rate of 50 mV s^−1^ to ensure reproducible, electrochemically active electrodes.

Cyclic voltammetry (CV) and chronoamperometry were performed using either an IPS Jaissle Potentiostat-Galvanostat PGU 10 V–100 mA, an IPS Jaissle Potentiostat-Galvanostat 1030 PC-T or an Ivium Vertex.One.EIS potentiostat. While CV experiments were conducted in one-compartment cells with a GC disc WE, electrolysis experiments were performed in a two-compartment cell separated by a Nafion membrane 117 (Alfa Aesar) using a GC plate WE. For both measurement types, a Pt CE and an Ag/AgCl RE were used. CV experiments in organic solvents were performed using an Ag/AgCl quasi-reference electrode, calibrated against ferrocene (Sigma Aldrich). In contrast, electrochemical measurements in aqueous media were conducted using a commercial Ag/AgCl (3 M KCl) (BASi) reference electrode.

Onset potentials (*E*_onset_) were determined using the intersection between the baseline and the tangent in the half-step of the signal current. Half-step potentials (*E*_p/2_) were determined as the potential at which the signal current equals half of the baseline corrected peak current.

The electrolyte solution of 0.1 M NaOH with a pH of 13 was prepared by dissolution of the respective amount of NaOH pellets (Merck) in 18 MΩ water. CV measurements in organic media were performed using a 0.1 M solution of tetrabutylammonium hexafluorophosphate (TBAPF_6_, Sigma Aldrich) in acetonitrile (MeCN, Carl Roth). Before usage, 1-hydroxyanthraquinone (1-OH-AQ, TCI Chemicals), 2-hydroxyanthraquinone (2-OH-AQ, Activate Scientific), 1,2-dihydroxyanthraquinone (1,2-OH-AQ, Alfa Aesar) and 1,4-dihydroxyanthraquinone (1,4-OH-AQ, Sigma Aldrich) were further purified by sublimation. Riboflavin (RF, Sigma Aldrich), sodium anthraquinone-2-sulfonate monohydrate (AQS, TCI Chemicals), Alizarin Red S sodium salt (ARS, Alfa Aesar) and unsubstituted Anthraquinone (AQ, Sigma Aldrich) were used as received.

Before each CV, the cells were purged with nitrogen (N_2_) for 1 h or O_2_ for 30 min to achieve saturated conditions. CV experiments in organic media were performed in a glove box under a nitrogen atmosphere. For all CV experiments, a scan rate of 200 mV s^−1^ was applied. Prior to each chronoamperometric measurement, both compartments were flushed with N_2_ and O_2_ for 30 min, and CV results were recorded to ensure saturated conditions. Electrolysis was performed at a constant potential of −0.25 V *vs.* SHE for 6 h. During the experiment, 100 μL aliquots were removed after 0, 1, 2, 4 and 6 h.

The amount of H_2_O_2_ produced during chronoamperometry was quantified spectroscopically according to a method reported by Apaydin *et al.*^53^ and Su *et al.*^54^ using 4-nitrobenzeneboronic acid (*p*-NBBA). Thus, a 4 mM solution of *p*-NBBA (Alfa Aesar) in dimethyl sulfoxide (VWR) was prepared and mixed in a volumetric ratio of 1 : 1 with a 150 mM carbonate buffer NaHCO_3_/Na_2_CO_3_ (Fluka & Sigma Aldrich), adjusted to a pH value of 9. The mixture was filtered through a syringe filter (Chromafil RC-45/15 MS, Macherey-Nagel) and subsequently added to the samples. After 36 min, the absorption spectra were measured at a wavelength of 411 nm using a Thermo Scientific Multiskan GO spectrometer. The external calibration made from H_2_O_2_ standard solutions (Merck), and the measured absorbance spectrum can be seen in Fig. S3.†

Unless specified otherwise, all potentials stated in this work refer to V *vs.* SHE.

### 2.3 UV-vis spectroscopy

A Varian Cary 3G UV-visible spectrophotometer was used to record the UV-vis absorption spectra of the substances dissolved in pure acetonitrile or 0.1 M NaOH. The UV-vis spectra were measured in a quartz cuvette (path length of 10 mm) in a 250 to 800 nm wavelength range.

## 3. Results and discussion

**Fig. 1:**
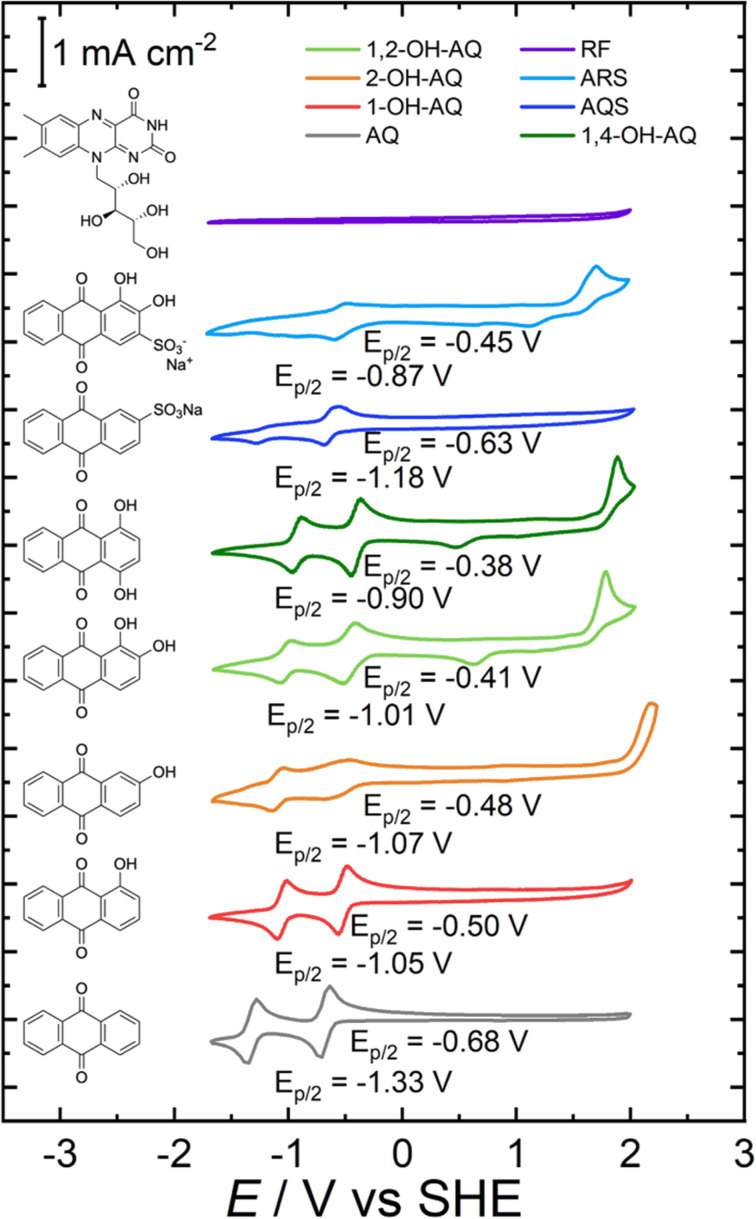
Cyclic voltammetry of the AQ molecules in 0.1 M TBAPF_6_ in MeCN solutions at a scan rate of 200 mV s^−1^. All compounds were investigated at a concentration of 1 mM, except for AQS (0.5 mM) and RF (0.05 mM).

The electrochemical properties of the target molecules were investigated by CV in the organic, aprotic solvent MeCN as depicted in Fig. 1.

With respect to the unsubstituted AQ, a clear effect of positively shifted reduction potentials upon substitution with hydroxy groups is observed in aprotic solution. Substitution with a second OH-group even leads to a further positive shift in reduction potentials, with the 1,4-substitution pattern (*para*-position) possessing the strongest influence. This effect is caused by intramolecular hydrogen bondings and is absent in the case of alkoxy-substituents,^55^ and is already well-described for aprotic solvents in previous experimental and computational studies.^56–58^ In contrast to the AQ derivatives, RF did not show remarkable redox features in organic, aprotic solvents, mainly attributed to the low solubility of the highly polar RF structure. As the electrocatalytic O_2_ to H_2_O_2_ reduction reaction is known to be most efficient for certain material classes in alkaline solutions,^40^ homogeneous solutions of the compounds shown in Fig. 1 were also examined in 0.1 M NaOH for their electrocatalytic performance. The choice of hydroxy-substituted AQs thereby combines two benefits. In addition to the higher H_2_O_2_ catalysis performance under alkaline conditions,^40^ the solubility of the hydroxy derivatives in aqueous, alkaline solution is sufficient for electrochemical investigations. Due to this reason, only hydroxy derivatives were chosen for this study alongside RF, which is known to be a catalyst for the ORR.^47,49,52^ The colour appearance of the material in solutions of 0.1 M NaOH and MeCN, as well as the respective UV-vis absorption spectra, can be seen in Fig. S4.†

**Fig. 2:**
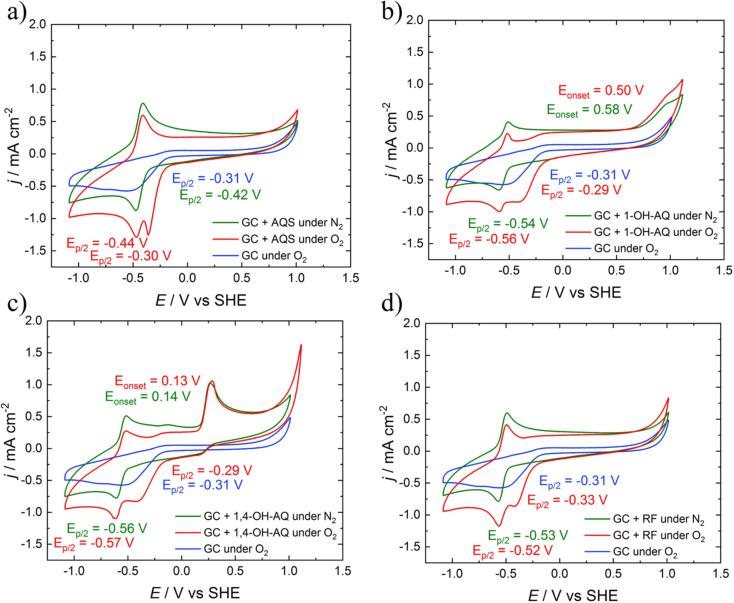
CV studies of a) AQS, b) 1-OH-AQ, c) 1,4-OH-AQ, and d) RF in 0.1 M NaOH recorded with a GC disc electrode at 200 mV s^−1^. All materials were studied in a concentration of 1 mM apart from 1-OH-AQ, which was studied in a 0.4 mM solution due to a lower solubility.

Fig. 2 depicts the CV curves of AQS, 1-OH-AQ, 1,4-OH-AQ and RF in 0.1 M NaOH under N_2_ and O_2_ saturated conditions together with O_2_ using a GC electrode in 0.1 M NaOH without the addition of any catalyst. The blue CV curves of GC in 0.1 M NaOH under O_2_ saturated conditions in Fig. 2 show the known electrocatalytic behaviour of GC, which overlaps with all AQ and RF reduction peaks. In the case of AQS under O_2_ in Fig. 2a, the resulting CV curve shows two close reduction peaks at half-step potentials of −0.30 and −0.44 V and no oxidation peak of AQS in the potential range examined. In the case of 1-OH-AQ and 1,4-OH-AQ under O_2_, Fig. 2b and c show reduction plateaus followed by reduction peaks at *E*_p/2_ of −0.56 V for 1-OH-AQ and −0.57 V for 1,4-OH-AQ, which is at the potential of the respective AQ species under N_2_ conditions. While 1-OH-AQ shows an oxidation feature at the border of the potential range investigated at an onset potential of 0.58 V, 1,4-OH-AQ possesses a clear and distinct oxidation peak at an onset potential of 0.13 V, which has an important influence on RRDE experiments to be discussed later. RF possesses a similar electrochemical behaviour to AQS with no oxidation peak. The CV graphs of 2-OH-AQ, 1,2-OH-AQ and ARS can be found in Fig. S5.† Upon comparing the CV curves, 2-OH-AQ behaves similarly to 1-OH-AQ while 1,2-OH-AQ and ARS behave similarly to 1,4-OH-AQ. The reduction potential values and oxidation onset potentials of all the materials studied are summarised in Table 1.

**Table 1:** Half step potentials *E*_p/2_ and onset potentials for the oxidation of pristine GC and 1 mM AQ derivatives (except 0.4 mM solution of 1-OH-AQ) in 0.1 M NaOH

Compound	*E*_p/2 reduction_/V *vs.* SHE		*E*_onset oxidation_/V *vs.* SHE	
	N_2_	O_2_	N_2_	O_2_
—	—	−0.31	—	—
1-OH-AQ	−0.54	−0.29 and −0.56	0.58	0.50
2-OH-AQ	−0.59	−0.28, −0.49 and −0.64	0.78	0.66
1,2-OH-AQ	−0.54 and −0.71	−0.24, −0.56 and −0.70	0.21 and 0.87	0.20 and 0.83
1,4-OH-AQ	−0.56	−0.29 and −0.57	0.14	0.13
AQS	−0.42	−0.30 and −0.44	—	—
ARS	−0.53 and −0.72	−0.28, −0.56 and −0.74	0.29	0.29
RF	−0.53	−0.33 and −0.52	—	—

As can be seen, in all cases studied, the GC ORR feature overlaps with the reduction peaks of the materials. The red curves with the catalytic compounds and O_2_ present in Fig. 2 indicate a significant increase in the electrocatalytic reductive current at potentials significantly more positive than the reduction of the respective compound under oxygen-free conditions. Using the mathematical approach, we have recently reported for RRDE studies of homogeneous solutions,^42^ we investigated the comparative application of this method to solutions of all the selected materials in the present study.

**Fig. 3:**
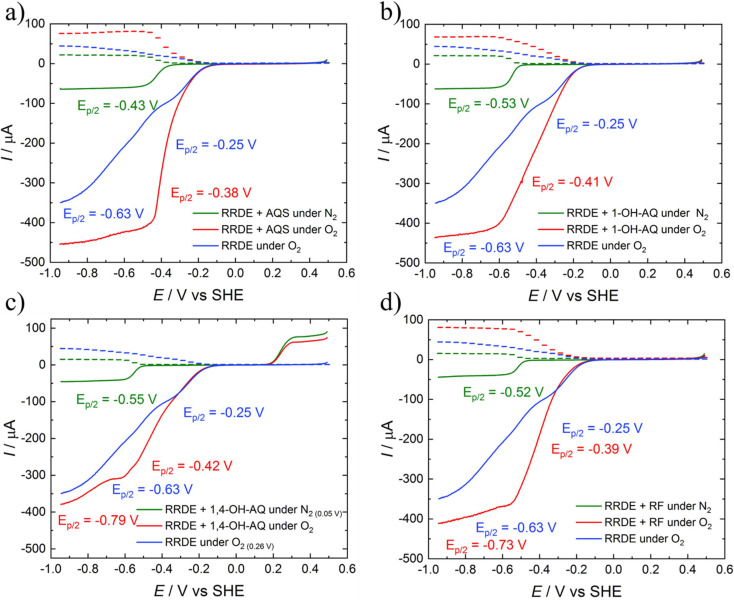
LSV graphs with ring (dashed line) and disc (solid line) currents of the 0.4 mM solutions of a) AQS, b) 1-OH-AQ, c) 1,4-OH-AQ, and d) RF in 0.1 M NaOH recorded with a GC/Pt RRDE electrode at 10 mV s^−1^. The graphs shown were recorded at 900 rpm and a ring potential of 0.26 V unless stated elsewise.

The LSV curves of AQS, 1-OH-AQ and 1,4-OH-AQ and RF are depicted in Fig. 3. The LSV curves in Fig. 3 and S6† reveal the electrocatalytic effect of the molecules even more plainly than the CV graphs in Fig. 2. Up to a certain starting point around −0.2 V, no effect is observed, as the line for O_2_ saturated conditions without any catalyst (blue) is identical to the line with the catalyst (red). Also, at very negative potentials, the molecule under O_2_ shows current values of the sum of 0.1 M NaOH under O_2_ (blue) and the compound dissolved in 0.1 M NaOH under N_2_ (green). However, in between those extrema, areas with significantly increased current going beyond the sum of the individual components are observed, especially close to the onset of the reduction.

**Fig. 4:**
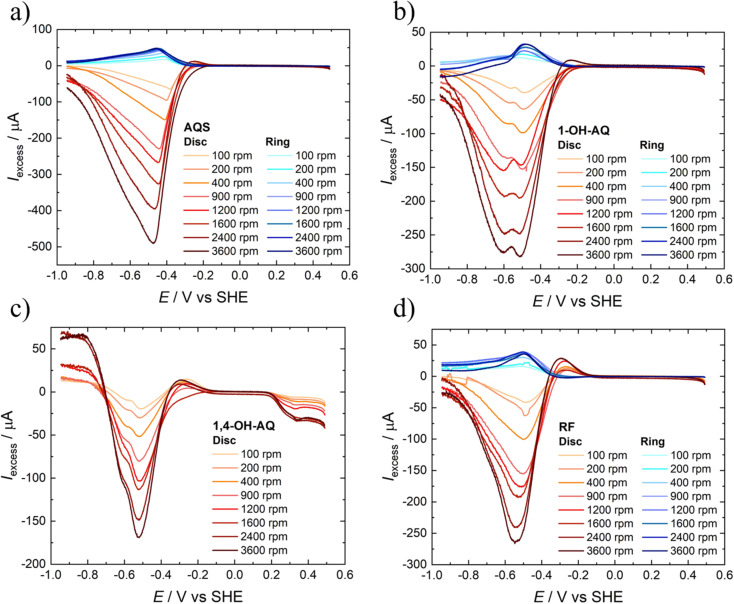
Excess current graphs of the 0.4 mM solutions of a) AQS, b) 1-OH-AQ, c) 1,4-OH-AQ, and d) RF in 0.1 M NaOH recorded with a GC/Pt RRDE electrode at 10 mV s^−1^.

With the help of eqn (3), for each catalyst material studied, excess currents for the ring and disc were calculated at all rotation speeds. Only for the two compounds exhibiting a very negative oxidation potential for the ring analyses, namely 1,2-OH-AQ and 1,4-OH-AQ, the excess currents were only determined for the disc. These excess currents are depicted in Fig. 4.

The excess current curves of AQS in Fig. 4a show a steep reductive current increase at around −0.4 V, which is qualitatively the potential range observed in Fig. 3a, where the AQS/O_2_ curve shows more current than the GC/O_2_ conditions. With a slight cathodic delay, the excess ring currents also show a less pronounced but similar peak behaviour, which is in accordance with the previous study.^42^ In contrast to AQS, 1-OH-AQ in Fig. 4b shows not only one reductive peak in the disc excess current curves but two peaks. While the first reductive peak also has a corresponding oxidative peak in the ring excess current curves, the second reductive peak lacks any feature in the ring currents. Comparing Fig. 4a and b with the onsets of the AQ/N_2_ reduction features in the LSV curves in Fig. 3a and b suggests that the second reductive peak corresponds to the reduction of 1-OH-AQ. As this feature is strongly pronounced in the disc excess currents, these results suggest an increased amount of electrochemically active 1-OH-AQ species in the vicinity of the electrode, most likely due to the electrocatalytic cycling within the time frame of the measurement.

Interestingly at first glance, RF in Fig. 4d shows a similar behaviour to AQS. However, an oxidative excess current peak is observed prior to the strong reductive currents in the disc excess currents. This anti-catalytic behaviour may be attributed to an activation pathway prior to the actual electrocatalytic process. Further excess current plots can be found in Fig. S7.† Thereby, examples of two or more reductive disc current peaks, as well as ARS with two reductive and two corresponding oxidative peaks, are illustrated. Based on the formula in eqn (2) for expressing the ratio of disc and ring currents with an efficiency, according to Wielend *et al.*^42^ excess efficiencies were calculated, as depicted in Fig. S8.† In addition, the diffusion coefficients of the compounds determined from CV studies are listed in Table S1.† The interpretation of these data is the focus of ongoing research.

**Fig. 5:**
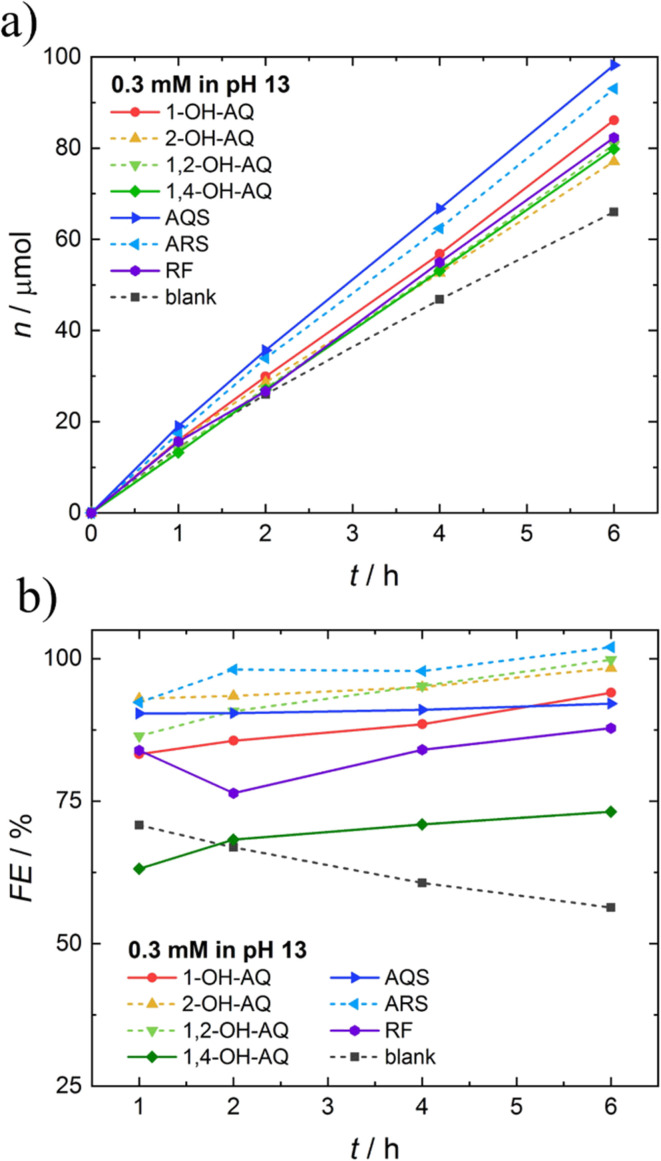
a) Comparison of moles of H_2_O_2_ produced using 0.3 mM homogeneously dissolved AQ molecules and RF. b) Comparison of faradaic efficiencies (FE) of the AQ derivatives and RF.

From the excess current curves, important catalytic values were extracted for all materials investigated and are depicted in Table 2. In order to draw conclusions from the electrocatalytic values in Table 2 to establish electrocatalytic parameters like productivity and selectivity, chronoamperometric electrolysis of 0.3 mM solutions of the compounds was performed over 6 h in 0.1 M NaOH, and the amount of H_2_O_2_ produced was quantified. As this colorimetric H_2_O_2_ detection method is based on the colour change of 4-nitrophenyl boronic acid, as demonstrated in Fig. S3,† possible interferences of the homogeneously dissolved electrocatalysts had to be considered. As illustrated in Fig. S9,† no distracting absorption peaks originating from the catalysts were observed during the H_2_O_2_ quantification, as also recently demonstrated,^52^ except for 1,4-OH-AQ. Hereby, the H_2_O_2_ quantification was not altered. Still, a steady decrease in absorption around 560 and 590 nm was observed, which was identified using Fig. S4† as the characteristic bands of the deprotonated 1,4-OH-AQ species. Furthermore, during the electrolysis of 1,4-OH-AQ, a colour change of the electrolyte solution was observed from dark violet to a paler colour. The same trend from a violet colour to a pale pink colour transition was even observed without applying any potential over two weeks under ambient conditions. A controlled investigation of this colour change depicted in Fig. S10† proved a steady degradation of 1,4-OH-AQ with air, which is already reported in the literature.^59^Fig. 5 depicts the moles of H_2_O_2_ produced *via* O_2_ reduction and the respective FE of the electrocatalytic processes. The chronoamperometric current *vs.* time plots for all molecules can be found in Fig. S11.†

One important conclusion from Fig. 5 is that also a bare GC electrode in 0.1 M NaOH solution (black dotted line) produces mentionable amounts of H_2_O_2_ at moderate average FEs of around 55%. Due to this fact, this significant background electrocatalytic activity has to be carefully considered when discussing molecular electrocatalytic activities, as we have recently demonstrated and emphasised.^23,52,55^ This electroactivity of pristine GC for O_2_ reduction is also present in the CV and LSV studies already discussed in Fig. 2 and 3. The exact values for the amount of H_2_O_2_ produced and mean FE can be found in Table 3.

**Table 2:** Summary of the determined maxima of the excess currents (*I*_excess,max_) with the corresponding potentials (*E*(*I*_excess,max_)), the onset potentials of the catalytic regions (*E*_onset_(*I*_excess_)), the potentials at the efficiency maxima (*E*(Efficiency_max_)) and the excess charges (*Q*_max_)

Compound	*I*_excess,max_/μA	*E*(*I*_excess,max_)/V *vs.* SHE	*E*_onset_(*I*_excess_)/V *vs.* SHE	*E*(Efficiency_max_)/V *vs.* SHE	*Q*_excess_/mC
1-OH-AQ	−283 and −277	−0.51 and −0.60	−0.31	−0.53	−10.8
2-OH-AQ	−296 and −275	−0.56 and −0.69	−0.34	−0.69	−11.3
1,2-OH-AQ	−129 and −132	−0.59 and −0.81	−0.17	—	−6.0
1,4-OH-AQ	−169	−0.52	−0.37	—	−2.4
AQS	−491	−0.47	−0.31	−0.42	−16.9
ARS	−265	−0.47	−0.30	—	−6.2
RF	−266	−0.55	−0.37	−0.53	−7.0

**Table 3:** Mean FE and total amount of H_2_O_2_ produced during electrolysis

Compound	FE_mean_/%	*n*_H_2_O_2__/μmol
—	62	66.0
1-OH-AQ	89	86.1
2-OH-AQ	96	77.1
1,2-OH-AQ	95	80.8
1,4-OH-AQ	70	79.8
AQS	91	98.2
ARS	98	93.1
RF	84	82.3

Despite this background activity, Table 3 demonstrates an increased electrocatalytic effect upon addition of all AQ derivatives and RF by larger quantities of H_2_O_2_ produced at strongly elevated FEs. Thus, all compounds show an increase in both productivity and selectivity. The fact that hydroxy-AQ derivatives show an enhanced electrocatalytic ORR activity was already demonstrated in 1997 in aprotic solvents.^60^ Interestingly, AQ derivatives carrying a sulfonate group show the highest productivity, at 98.2 μmol (AQS) and 93.1 μmol (ARS) of H_2_O_2_ produced. However, 2-hydroxy derivatives show the highest selectivity, with FE values of 96% (2-OH-AQ) and 95% (1,2-OH-AQ). Overall, ARS, which carries both sulfonate- and hydroxy-groups, demonstrates a high productivity and selectivity.

**Fig. 6:**
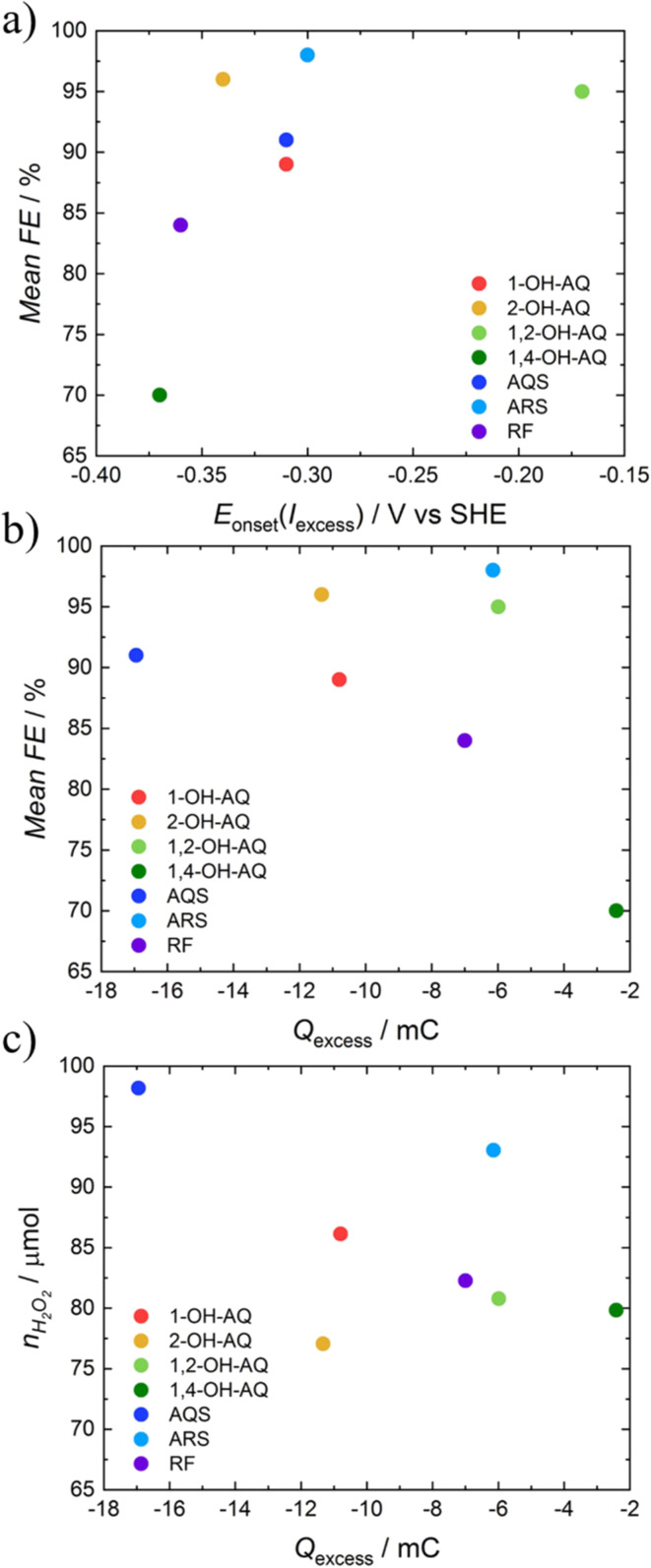
Comparison of the mean FE with the a) onset potential of the excess current (*E*_onset_(*I*_excess_)) and b) excess charge (*Q*_excess_) as well as c) comparison of the amount of substance of H_2_O_2_ produced during the electrolysis (*n*_H_2_O_2__) with the *Q*_excess_ of AQs and RF.

A comparison of the characteristic electrocatalytic values of the RRDE with the chronoamperometric electrolysis method can be found in Fig. 6. As seen in Fig. 6, no ultimate quantitative correlation can be found. However, certain qualitative behaviour can be observed. A lower onset potential of the excess current correlates with a higher FE in electrolysis experiments, which can be seen in Fig. 6a. Furthermore, as depicted in Fig. 6b, a lower excess charge coincides with a lower FE. A clear trend can be found when comparing the total amount of H_2_O_2_ produced over 6 h with the excess charge found using the RRDE, as depicted in Fig. 6c. Here, a decrease in the excess current is accompanied by a decrease in the production of H_2_O_2_, which can be understood by the fact that both quantities are closely related to the O_2_ to H_2_O_2_ reduction reaction productivity.

Moreover, all comparisons show the poor performance of 1,4-OH-AQ, which may be linked to the decomposition of the compound during the electrolysis experiment and upon prolonged O_2_ exposure. This strong dependence of the position of the hydroxy groups on the electrocatalytic behaviour was also already observed earlier by the group of Richard Compton, where also 1,4-OH-AQ was identified as a less active catalyst.^61^ A comparison of the mean FE with the maximum excess current can be found in Fig. S12,† which shows a worse correlation than the excess charge in Fig. 6c.

## 4. Conclusions

In this study, homogeneously dissolved anthraquinone derivatives and riboflavin were investigated for their electrocatalytic capacity for O_2_ reduction using a new RRDE subtraction method. This method was compared with established chronoamperometric electrolysis experiments using a quantitative spectroscopic detection method for H_2_O_2_. Upon the addition of the electrocatalyst, a significant increase in the reductive current at more positive potentials was found. This increase in the reductive current and, thus, electrocatalytic activity was further visualised through the calculation of the excess current from the RRDE experiments.

The steep increase of the excess current was found around the potential of the reduction of the catalytic compound itself, mirrored by a ring current with a slight catalytic delay. However, the ring current could not be determined for compounds with a very negative oxidation potential, as they are interfering with the oxidation of H_2_O_2_ at the ring.

Chronoamperometric measurements reaffirmed the catalytic activity of all compounds investigated, showing an increase in both productivity and selectivity towards H_2_O_2_. A comparison of both methods yields qualitative trends, with an increase in the excess current correlating to an increased FE as well as a higher amount of H_2_O_2_ produced.

These results demonstrate the RRDE subtraction method as a valuable tool, complementary to chronoamperometric electrolysis, for comparing the electrocatalytic ability of various compounds in a fast and simple way.

## Author contributions

Angelina Kerschbaumer: investigation, visualisation, writing – original draft. Dominik Wielend: conceptualisation, investigation, methodology, project administration, writing – original draft. Elisabeth Leeb: investigation, visualisation, writing – original draft. Corina Schimanofsky: investigation, writing – original draft. Nadine Kleinbruckner: investigation, writing – original draft. Helmut Neugebauer: validation, writing – review & editing. Mihai Irimia-Vladu: resources. Niyazi Serdar Sariciftci: funding acquisition, supervision, writing – review & editing.

All authors have approved the final version of the manuscript.

## Conflicts of interest

The authors have declared, they have no conflict of interest regarding this content.

## Supplementary Material

CY-013-D2CY01744J-s001

## References

[cit1] Faunce T. A., Lubitz W., Rutherford A. W., MacFarlane D., Moore G. F., Yang P., Nocera D. G., Moore T. A., Gregory D. H., Fukuzumi S., Yoon K. B., Armstrong F. A., Wasielewski M. R., Styring S. (2013). Energy Environ. Sci..

[cit2] Lewis N. S., Nocera D. G. (2007). Proc. Natl. Acad. Sci. U. S. A..

[cit3] Kalyanasundaram K., Graetzel M. (2010). Curr. Opin. Biotechnol..

[cit4] She Z. W., Kibsgaard J., Dickens C. F., Chorkendorff I., Nørskov J. K., Jaramillo T. F. (2017). Science.

[cit5] Häupler B., Wild A., Schubert U. S. (2015). Adv. Energy Mater..

[cit6] Han C., Li H., Shi R., Zhang T., Tong J., Li J., Li B. (2019). J. Mater. Chem. A.

[cit7] Werner D., Apaydin D. H., Portenkirchner E. (2018). Batteries Supercaps.

[cit8] Werner D., Apaydin D. H., Wielend D., Geistlinger K., Saputri W. D., Griesser U. J., Drazevic E., Hofer T. S., Portenkirchner E. (2021). J. Phys. Chem. C.

[cit9] Campos-Martin J. M., Blanco-Brieva G., Fierro J. L. G. (2006). Angew. Chem., Int. Ed..

[cit10] Sehrish A., Manzoor R., Dong K., Jiang Y., Lu Y. (2019). Chemical Reports.

[cit11] Ciriminna R., Albanese L., Meneguzzo F., Pagliaro M. (2016). ChemSusChem.

[cit12] Fukuzumi S., Yamada Y. (2016). ChemElectroChem.

[cit13] Disselkamp R. S. (2008). Energy Fuels.

[cit14] Bu Y., Wang Y., Han G. F., Zhao Y., Ge X., Li F., Zhang Z., Zhong Q., Baek J. B. (2021). Adv. Mater..

[cit15] Vikkisk M., Kruusenberg I., Joost U., Shulga E., Kink I., Tammeveski K. (2014). Appl. Catal., B.

[cit16] Ikeda T., Hou Z., Chai G. L., Terakura K. (2014). J. Phys. Chem. C.

[cit17] Lu Y., Li X., Kaliyaraj A., Kumar S., Compton R. G. (2022). ACS Catal..

[cit18] Sun Y., Sinev I., Ju W., Bergmann A., Dresp S., Kühl S., Spöri C., Schmies H., Wang H., Bernsmeier D., Paul B., Schmack R., Kraehnert R., Roldan Cuenya B., Strasser P. (2018). ACS Catal..

[cit19] Jakešová M., Apaydin D. H., Sytnyk M., Oppelt K., Heiss W., Sariciftci N. S., Głowacki E. D. (2016). Adv. Funct. Mater..

[cit20] Murata T., Kotsuki K., Murayama H., Tsuji R., Morita Y. (2019). Commun. Chem..

[cit21] Khomenko V. G., Barsukov V. Z., Katashinskii A. S. (2005). Electrochim. Acta.

[cit22] Wu A., Venancio E. C., MacDiarmid A. G. (2007). Synth. Met..

[cit23] Rabl H., Wielend D., Tekoglu S., Seelajaroen H., Neugebauer H., Heitzmann N., Apaydin D. H., Scharber M. C., Sariciftci N. S. (2020). ACS Appl. Energy Mater..

[cit24] Hu J., Li S. S., Li J. F., Wang Y. L., Zhang X. Y., Chen J. B., Li S. Q., Gu L. N., Chen P. (2022). Chem. Eng. J..

[cit25] Mitraka E., Gryszel M., Vagin M., Jafari M. J., Singh A., Warczak M., Mitrakas M., Berggren M., Ederth T., Zozoulenko I., Crispin X., Głowacki E. D. (2019). Adv. Sustainable Syst..

[cit26] Vagin M., Gueskine V., Mitraka E., Wang S., Singh A., Zozoulenko I., Berggren M., Fabiano S., Crispin X. (2021). Adv. Energy Mater..

[cit27] Vaik K., Sarapuu A., Tammeveski K., Mirkhalaf F., Schiffrin D. J. (2004). J. Electroanal. Chem..

[cit28] Vaik K., Mäeorg U., Maschion F. C., Maia G., Schiffrin D. J., Tammeveski K. (2005). Electrochim. Acta.

[cit29] Sarapuu A., Helstein K., Vaik K., Schiffrin D. J., Tammeveski K. (2010). Electrochim. Acta.

[cit30] Dalle K. E., Warnan J., Leung J. J., Reuillard B., Karmel I. S., Reisner E. (2019). Chem. Rev..

[cit31] Whang D. R. (2020). Nano Convergence.

[cit32] Wielend D., Salinas Y., Mayr F., Bechmann M., Yumusak C., Neugebauer H., Brüggemann O., Sariciftci N. S. (2021). ChemElectroChem.

[cit33] Li Q., Batchelor-McAuley C., Lawrence N. S., Hartshorne R. S., Compton R. G. (2011). Chem. Commun..

[cit34] Batchelor-McAuley C., Dimov I. B., Aldous L., Compton R. G. (2011). Proc. Natl. Acad. Sci. U. S. A..

[cit35] Chowdhury P., Fortin P., Suppes G., Holdcroft S. (2016). Macromol. Chem. Phys..

[cit36] Sun J., Wu Y. (2020). Angew. Chem., Int. Ed..

[cit37] Mirkhalaf F., Tammeveski K., Schiffrin D. J. (2004). Phys. Chem. Chem. Phys..

[cit38] Šljukić B., Banks C. E., Compton R. G. (2005). J. Iran. Chem. Soc..

[cit39] Berl E. (1939). Trans. Electrochem. Soc..

[cit40] Zhang H., Lin C., Sepunaru L., Batchelor-McAuley C., Compton R. G. (2017). J. Electroanal. Chem..

[cit41] Hossain M. S., Tryk D., Yeager E. (1989). Electrochim. Acta.

[cit42] Wielend D., Neugebauer H., Sariciftci N. S. (2021). Electrochem. Commun..

[cit43] Dalton F. (2016). Electrochem. Soc. Interface.

[cit44] Perry S. C., Pangotra D., Vieira L., Csepei L. I., Sieber V., Wang L., Ponce de León C., Walsh F. C. (2019). Nat. Rev. Chem..

[cit45] Tammeveski K., Kontturi K., Nichols R. J., Potter R. J., Schiffrin D. J. (2001). J. Electroanal. Chem..

[cit46] Goyal A., Marcandalli G., Mints V. A., Koper M. T. M. (2020). J. Am. Chem. Soc..

[cit47] Kormányos A., Hossain M. S., Foss F. W., Janáky C., Rajeshwar K. (2016). Catal. Sci. Technol..

[cit48] Kormányos A., Hossain M. S., Ghadimkhani G., Johnson J. J., Janáky C., de Tacconi N. R., Foss F. W., Paz Y., Rajeshwar K. (2016). Chem. – Eur. J..

[cit49] Zhang J., Chi Q., Dong S., Wang E. (1996). J. Chem. Soc., Faraday Trans..

[cit50] Richtar J., Heinrichova P., Apaydin D. H., Schmiedova V., Yumusak C., Kovalenko A., Weiter M., Sariciftci N. S., Krajcovic J. (2018). Molecules.

[cit51] Richtar J., Ivanova L., Whang D. R., Yumusak C., Wielend D., Weiter M., Scharber M. C., Kovalenko A., Sariciftci N. S., Krajcovic J. (2021). Molecules.

[cit52] Leeb E., Wielend D., Schimanofsky C., Sariciftci N. S. (2022). Electrochem. Sci. Adv..

[cit53] Apaydin D. H., Seelajaroen H., Pengsakul O., Thamyongkit P., Sariciftci N. S., Kunze-Liebhäuser J., Portenkirchner E. (2018). ChemCatChem.

[cit54] Su G., Wei Y., Guo M. (2011). Am. J. Anal. Chem..

[cit55] Wielend D., Vera-Hidalgo M., Seelajaroen H., Sariciftci N. S., Pérez E. M., Whang D. R. (2020). ACS Appl. Mater. Interfaces.

[cit56] Gallmetzer J. M., Kröll S., Werner D., Wielend D., Irimia-Vladu M., Portenkirchner E., Sariciftci N. S., Hofer T. (2022). Phys. Chem. Chem. Phys..

[cit57] Shamsipur M., Siroueinejad A., Hemmateenejad B., Abbaspour A., Sharghi H., Alizadeh K., Arshadi S. (2007). J. Electroanal. Chem..

[cit58] Schimanofsky C., Wielend D., Kröll S., Lerch S., Werner D., Gallmetzer J. M., Mayr F., Neugebauer H., Irimia-Vladu M., Portenkirchner E., Hofer T. S., Sariciftci N. S. (2022). J. Phys. Chem. C.

[cit59] Itoh K., Kitade Y., Yatome C. (1998). Bull. Environ. Contam. Toxicol..

[cit60] Jeziorek D., Ossowski T., Liwo A., Dyl D., Nowacka M., Woźnicki W. (1997). J. Chem. Soc., Perkin Trans. 2.

[cit61] Mason J., Batchelor-McAuley C., Compton R. G. (2013). Phys. Chem. Chem. Phys..

