# Supplementary material for: How to use a rotating ring-disc electrode (RRDE) subtraction method to investigate the electrocatalytic oxygen reduction reaction?
Source: Catal Sci Technol. 2022 Dec 23;13(3):834–43. doi: 10.1039/d2cy01744j (PMC9900597; doi:10.1039/d2cy01744j)
Supplement: CY-013-D2CY01744J-s001 [file CY-013-D2CY01744J-s001.pdf]

Electronic Supplementary Material (ESI) for Catalysis Science & Technology.
This journal is © The Royal Society of Chemistry 2022

How to use rotating ring-disc electrode (RRDE) subtraction method to investigate the electrocatalytic oxygen reduction reaction?

Angelina Kerschbaumer§, Dominik Wielend§, Elisabeth Leeb, Corina Schimanofsky, Nadine Kleinbruckner, Helmut Neugebauer, Mihai Irimia-Vladu, Niyazi Serdar Sariciftci*

Linz Institute for Organic Solar Cells (LIOS), Institute of Physical Chemistry, Johannes Kepler University Linz, Altenberger Straße 69, 4040 Linz, Austria.

(*E-mail: elisabeth.leeb@jku.at)

Supporting Information

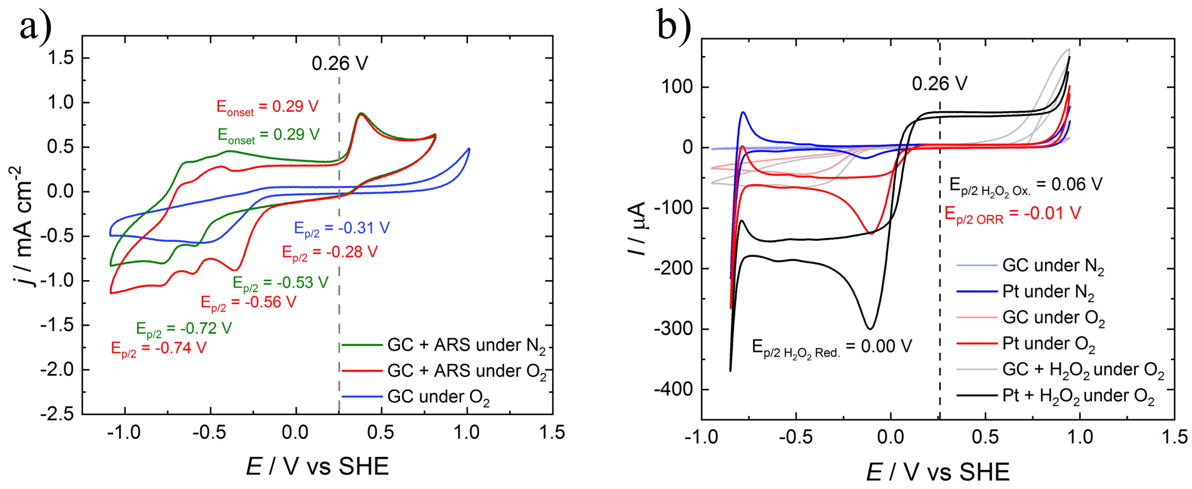

Figure S1: CVs for determining the ring potential with a) CV of 1 mM ARS in 0.1 M NaOH with the onset potential of its oxidation and b) CV curves of GC and Pt under N_2 and O_2 saturated conditions as well as of a 5 mM H_2O_2 solution in 0.1 M NaOH.

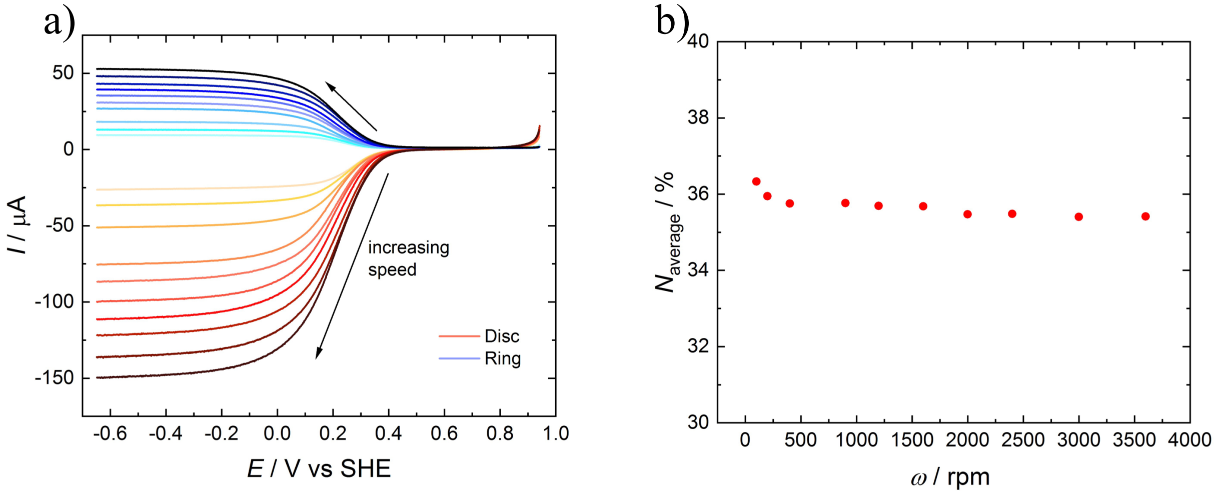

Figure S2: a) LSV of 1 mM $K_3[Fe(CN)_6]$ in 0.1 M NaOH using different rotational speeds under N_2 and b) average collection efficiency N at corresponding rotation rates.

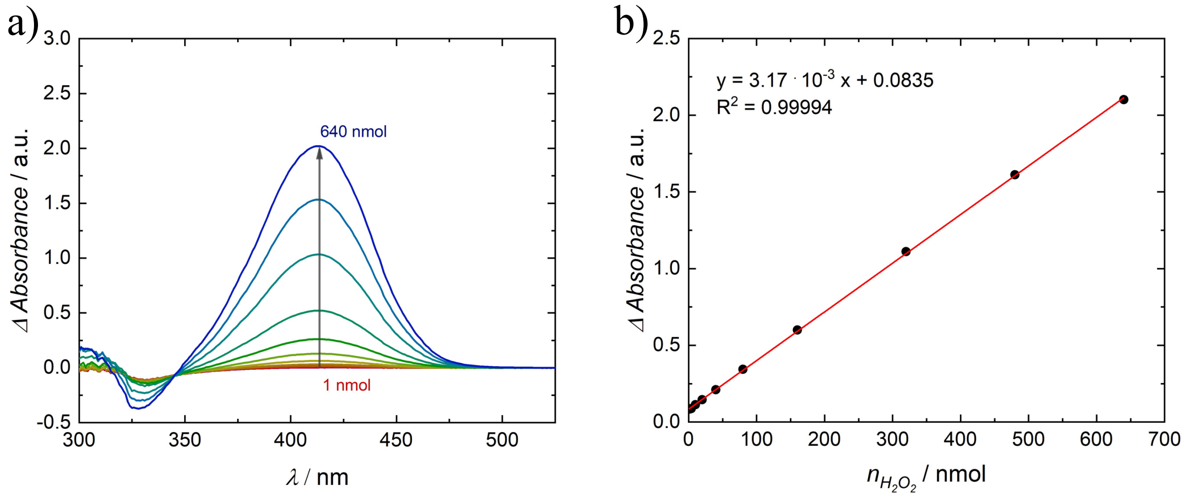

Figure S3: a) UV-VIS absorption spectra and b) calibration plot for the H_2O_2 quantification.

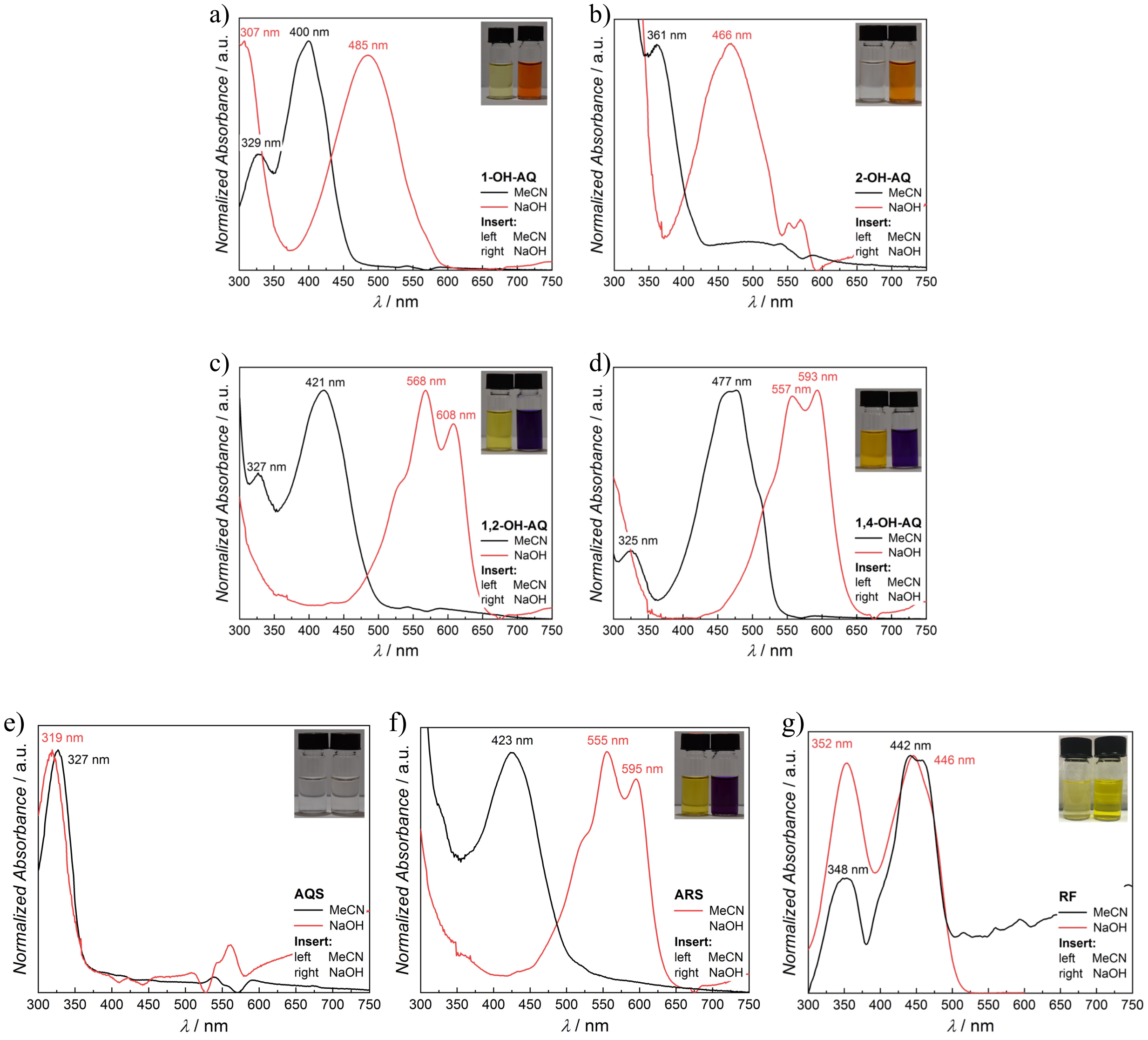

Figure S4: UV-Vis graphs of materials in MeCN and NaOH with picture of vials in the inset. a) 1-OH-AQ, b) 2-OH-AQ, c) 1,2-OH-AQ, d) 1,4-OH-AQ, e) AQS, f) ARS, and g) RF.

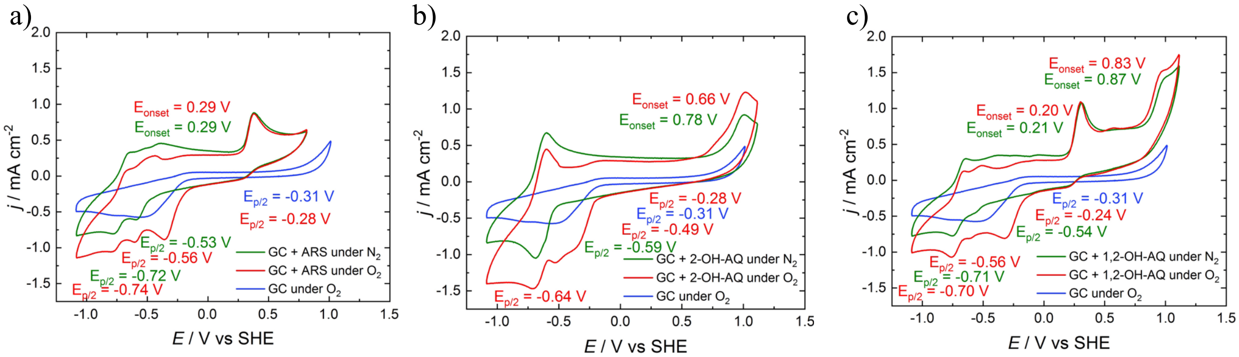

Figure S5: CV studies of 1 mM solutions of a) ARS, b) 2-OH-AQ, and c) 1,2-OH-AQ in 0.1 M NaOH recorded with a GC disc electrode at 200 mV s^{-1}.

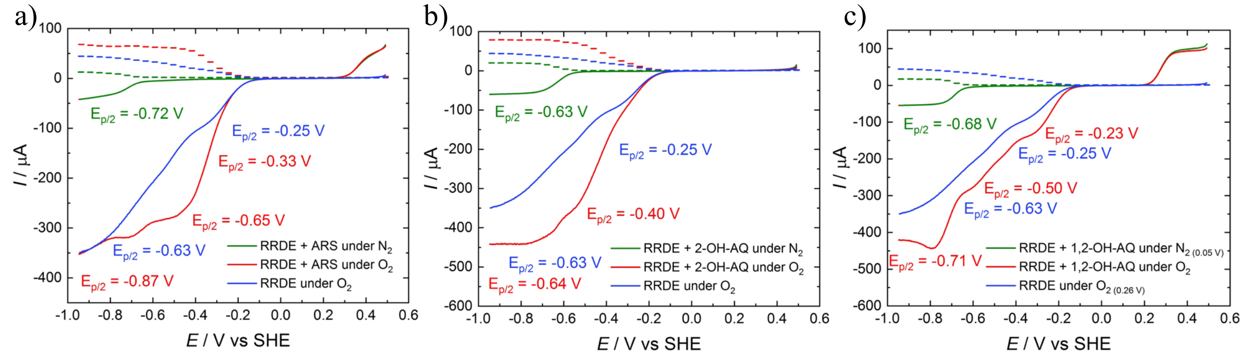

Figure S6: LSV graphs with ring (dashed line) and disc (solid line) current of 0.4 mM solutions of a) ARS, b) 2-OH-AQ and c) 1,2-OH-AQ in 0.1 M NaOH recorded with a GC/Pt RRDE electrode at 10 mV s^{-1}. The graphs shown were recorded at 900 rpm and a ring potential of 0.26 V unless stated otherwise.

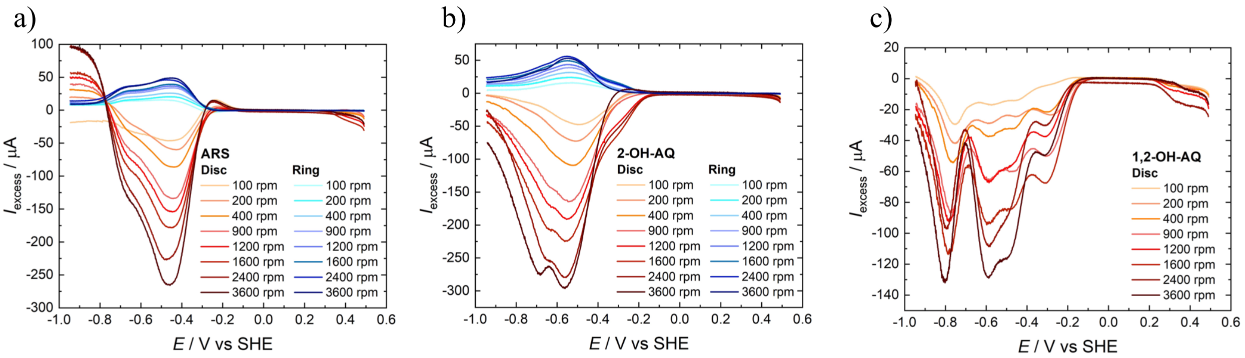

Figure S7: Excess current graphs of 0.4 mM solutions of a) ARS, b) 1-OH-AQ, and c) 1,2-OH-AQ in 0.1 M NaOH recorded with a GC/Pt RRDE electrode at 10 mV s^{-1}.

Table S1: Diffusion coefficients in 0.1 M NaOH and MeCN.

**Compounds**	**D_0 / cm s^{-1}**	
	**NaOH**	**MeCN**
1-OH-AQ	$8.73 \cdot 10^{-6}$	$1.47 \cdot 10^{-5}$
2-OH-AQ	$4.46 \cdot 10^{-6}$	$3.79 \cdot 10^{-6}$
1,2-OH-AQ	$2.30 \cdot 10^{-6}$	$1.15 \cdot 10^{-5}$
1,4-OH-AQ	$2.58 \cdot 10^{-6}$	$1.23 \cdot 10^{-5}$
AQS	$3.49 \cdot 10^{-6}$	$7.96 \cdot 10^{-6}$
ARS	$3.34 \cdot 10^{-7}$	$3.09 \cdot 10^{-6}$
RF	$2.20 \cdot 10^{-6}$	-
AQ	-	$1.92 \cdot 10^{-5}$

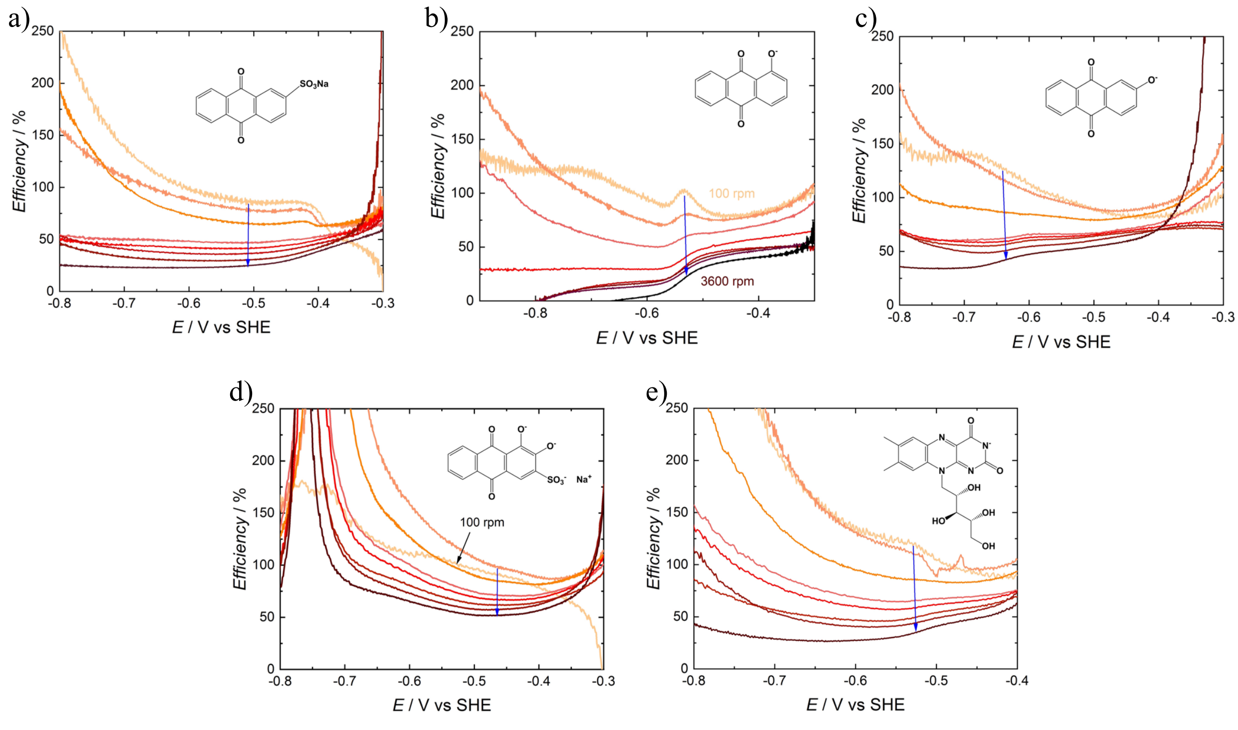

Figure S8: Excess efficiencies of a) AQS, b) 1-OH-AQ, c) 2-OH-AQ, d) ARS, and e) RF in 0.1 M NaOH.

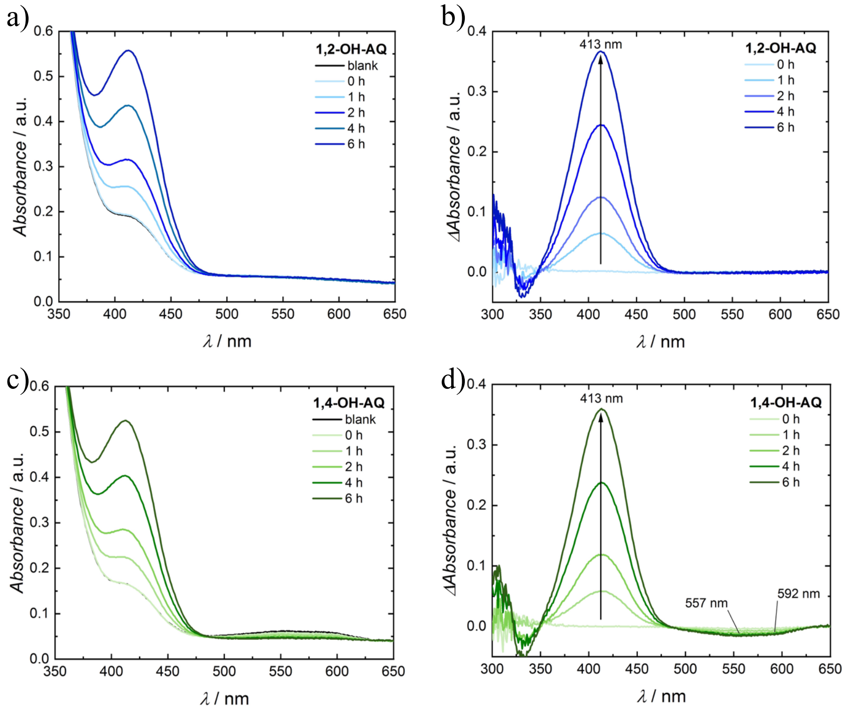

Figure S9: Comparison of the UV-Vis absorption spectra of H_2O_2 quantification of electrolysis over the course of 6 h as well as the Δabsorbance spectra using a, b) 1,2-OH-AQ and c, d) 1,4-OH-AQ as catalyst.

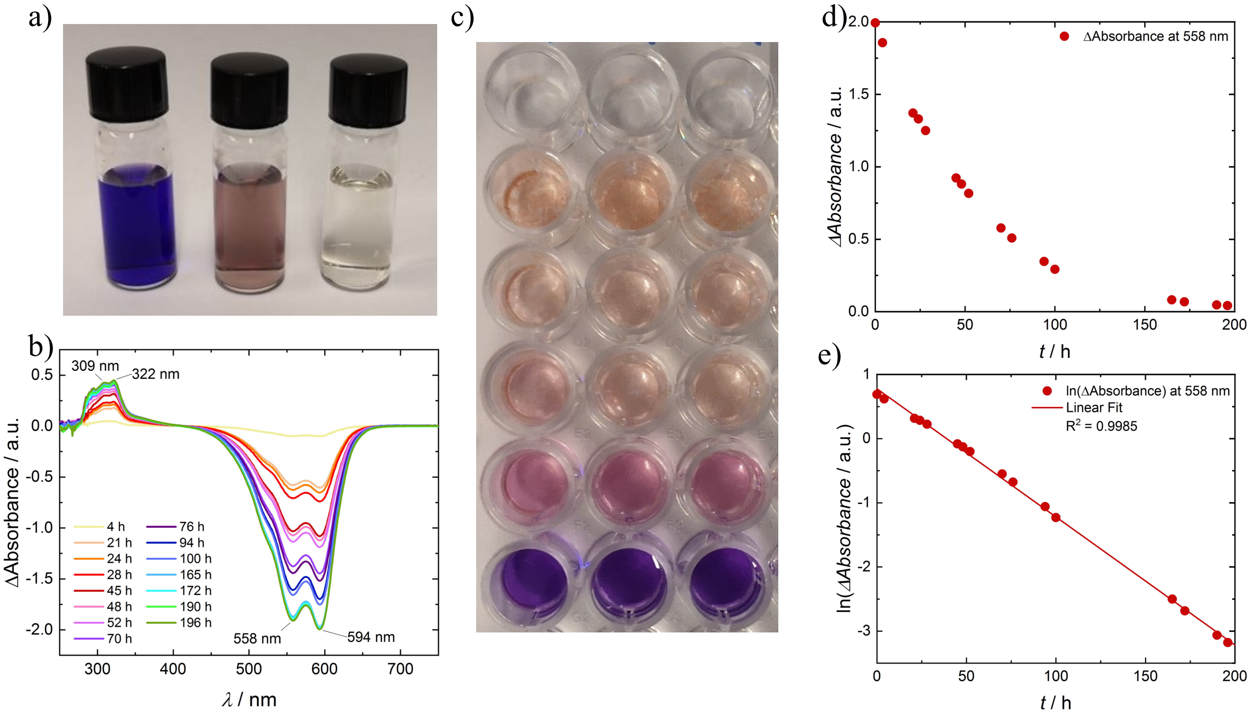

Figure S10: Study of colour disappearance of different 1,4-OH-AQ solutions in 0.1 M NaOH over time: a) picture of freshly made solution, as well as after 1 week and 2 months of storage, b) Δabsorbance spectra with increasing storage time c) picture of the well plate used for H_2O_2 detection, d) Δabsorbance at 558 nm against time and e) logarithm of Δabsorbance at 558 nm against time.

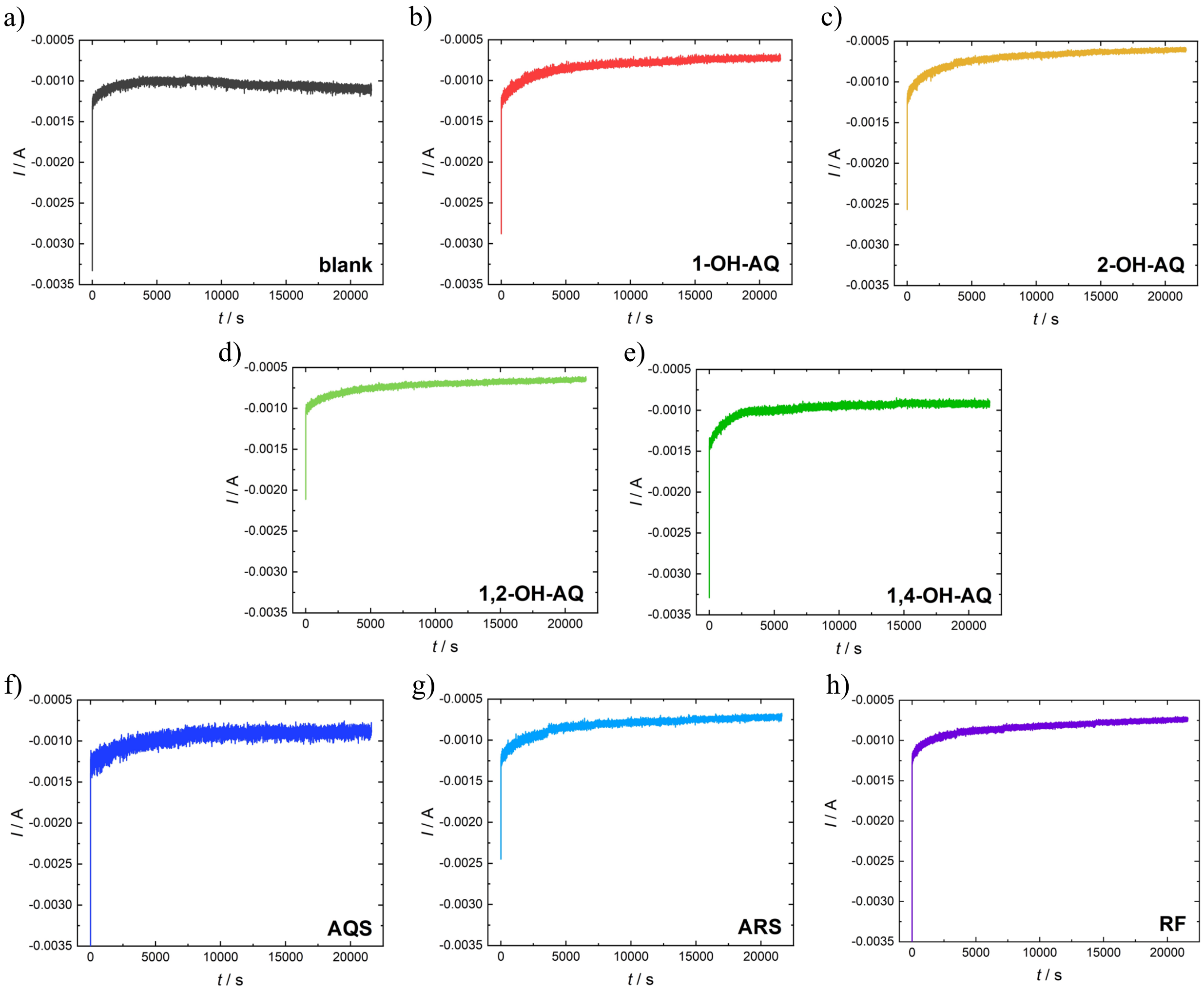

Figure S11: Current vs. time plots of all chronoamperometric measurements in 0.1 M NaOH at -400 mV. a) blank, 0.1 M NaOH without any catalyst added, b) 1-OH-AQ, c) 2-OH-AQ, d) 1,2-OH-AQ, e) 1,4-OH-AQ, f) AQS, g) ARS and h) RF.

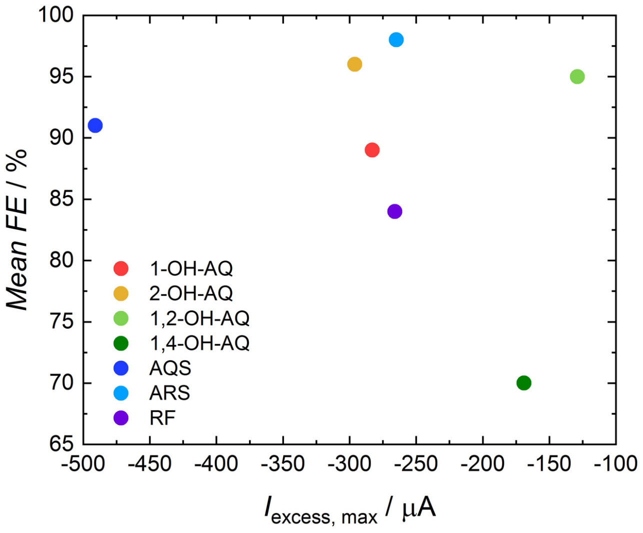

Figure S12: Mean FE over 6h of electrolysis in dependence of the maximum excess current.
